# Clinical guideline-guided prognostic models of functional disability after acute ischemic stroke: a retrospective cohort study

**DOI:** 10.1186/s12883-025-04432-w

**Published:** 2026-03-26

**Authors:** Mustapha Mohammed, Hadzliana Zainal, Siew Chin Ong, Balamurugan Tangiisuran, Fatimatuzzahra Abdul Aziz, Norsima Nazifah Sidek, Abubakar Sha’aban, Umar Idris Ibrahim, Surajuddeen Muhammad, Khadijat Muhammad, Irene Looi, Zariah A. Aziz

**Affiliations:** 1 Biomedical Research Center, QU Health, Qatar University, Doha, Qatar; 2 School of Pharmaceutical Sciences, Universiti Sains Malaysia, Pulau Pinang, Malaysia; 3 Clinical Research Centre, Hospital Sultanah Nur Zahirah, Terengganu, Malaysia; 4 Health and Care Research Wales Evidence Centre, Division of Population Medicine, School of Medicine, Cardiff University, Cardiff, UK; 5 Faculty of Pharmacy, Universiti Sultan Zainal Abidin, Terengganu, Malaysia; 6 Faculty of Veterinary Medicine, Ahmadu Bello University, Kaduna, Nigeria; 7 Department of Public Health, Iconic University, Sokoto, Nigeria; 8 Clinical Research Centre, Hospital Seberang Jaya, Seberang Jaya, Pulau Pinang, Malaysia

**Keywords:** Acute ischemic stroke, Prognostic model, Functional disability, Modified rankin scale, Validation

## Abstract

**Background:**

There is limited evidence on prognostic models that incorporate clinical guideline recommendations during the acute management of ischemic stroke. This study aimed to develop and validate clinical guideline-based prognostic models of functional disability following a first-ever acute ischemic stroke.

**Methods:**

A multicenter retrospective cohort study was conducted using data from 899 adult patients (aged 18 years or older) with a first-ever acute ischemic stroke, and enrolled in the Malaysian National Stroke Registry (NSR) between January 2009 and December 2019. The primary outcome was functional disability at three months post-discharge, defined as a modified Rankin Scale (mRS) score of 3 to 5. A multivariable logistic regression model was developed using random sampling data splitted into two cohorts: the development (*n* = 674; 75%) and the internal validation (*n* = 225; 25%). Model performance was evaluated by discrimination [area under the curve (AUC)] and calibration [Hosmer–Lemeshow (HL) test].

**Results:**

The final prognostic model showed factors associated with increased risk of functional disability include age [adjusted odds ratio (aOR) 1.02, 95% CI 1.00–1.04, *p* = 0.024], female gender [aOR 1.54, 95% CI 1.06–2.23, *p* = 0.024], and diabetes [aOR 1.66, 95% CI 1.15–2.40, *p* = 0.007]. While those factors associated with decreased risk of disability include posterior circulation infarct (POCI) [aOR 0.38, 95% CI 0.18–0.78, *p* = 0.009], and adherence to key performance indicators (KPIs); antiplatelet therapy [aOR 0.48, 95% CI 0.29–0.80, *p* = 0.005], lipid-lowering therapy [aOR 0.56, 95% CI 0.37–0.83, *p* = 0.004], stroke education [aOR 0.09, 95% CI 0.05–0.16, *p* < 0.001], and rehabilitation [aOR 0.43, 95% CI 0.29–0.64, *p* < 0.001]. The fitted model demonstrated good discrimination (AUC: 0.803; 95% CI, 0.78–0.83 for development, and 0.777; 95% CI, 0.75–0.81 for validation) and calibration (HL test: *p* = 0.473 for development, *p* = 0.967 for validation).

**Conclusion:**

A clinical guideline-guided prognostic model of functional disability following a first-ever acute ischemic stroke was successfully developed and validated, demonstrating strong performance and potential clinical relevance. This model can help clinicians predict stroke outcomes, guide informed clinical decision-making, and support the development of personalized stroke care.

## Introduction

Stroke remains the second leading cause of death and a leading cause of long-term disability globally [1]. The burden is disproportionately higher in low- and middle-income countries, particularly in Asia [2]. Ischemic stroke accounts for approximately 75% of all stroke cases [3], and requires timely and multifaceted clinical management. Early interventions aimed at restoring cerebral perfusion and minimizing neurological damage are critical to improving functional outcomes [4]. Post-stroke disability severity in the acute phase strongly predicts long-term recovery and prognosis [5]. Clinical practice guidelines (CPGs) offer evidence-based recommendations for the diagnosis, acute management, and secondary prevention of stroke [6–8].

In Malaysia, stroke ranks as the third leading cause of mortality and the second leading cause of disability, a trend expected to worsen with an aging population [9]. Data from the Malaysia National Stroke Registry (2009–2017) highlight appreciable gains in stroke outcomes at hospital discharge among first-ever stroke patients. Functional independence rose significantly from 23.3% in 2009 to 46.5% in 2017 [10]. In 2020, Malaysia released its latest guidelines for the management of acute ischemic stroke, which marked a milestone by introducing nine key performance indicators (KPIs) to evaluate and enhance the quality of stroke care [11–13]. These stroke care KPIs include thrombolytic therapy, antithrombotic therapy, dysphagia screening, deep vein thrombosis (DVT) prophylaxis, anticoagulation therapy for atrial fibrillation (AF), lipid-lowering therapy, stroke education, and rehabilitation. A previous study in Malaysia among patients with ischemic stroke reported that adherence to stroke care KPIs was suboptimal and associated with higher mortality [14].

Prognostic models serve as essential tools in providing individualized estimates of stroke outcomes [15]. Such models can support shared decision-making, optimize resource allocation, and facilitate personalized rehabilitation planning. However, existing models often lack stroke-specificity, exhibit limited external validity, and provide minimal utility for long-term functional outcomes [16]. Tools like the Framingham Stroke Risk Score [17] and QRISK [18], are widely used for cardiovascular risk assessment but are suboptimal for acute stroke prognostication. There have been reported limitations of the existing stroke prognostic risk scores in external populations [16, 19]. Evidence suggests that models relying solely on baseline clinical data before intervention struggle to predict post-stroke functional outcomes accurately [20].

Despite the proliferation of stroke prognostic research, which has yielded over 60 multivariable models and more than 190 candidate predictors, no consensus has emerged regarding a universally applicable model [21]. While models derived from large datasets, such as the Get With The Guidelines-Stroke (GWTG-Stroke) registry, demonstrate robust performance, they are still not generalizable to externally diverse healthcare systems and populations, such as those in Malaysia. A study from Malaysia established a validated prognostic model of mortality after a first-ever acute stroke in the Malaysian population [22]. Another study reported that functional disability, as measured by the mean Barthel Index (BI) scores, improved from discharge to three months post-discharge over time, with time, age, and stroke subtype identified as significant prognostic factors [23]. While the BI is more useful in rehabilitation settings, the modified Rankin scale (mRS) is generally considered a better tool for assessing overall functional disability and comparing outcomes. Therefore, this study aims to develop and validate a prognostic model for predicting functional disability in patients with first-ever acute ischemic stroke in Malaysia.

## Methods

### Study design

This multicenter retrospective cohort study aimed to develop and validate a guideline-based predictive model for functional disability following a first-ever acute ischemic stroke in Malaysia. The study included cases recorded from 1 st January 2009 to 31 st December 2019. Methodological reporting adhered to the Guidelines for Reporting of Statistics for Clinical Research, including the Strengthening the Reporting of Observational Studies in Epidemiology (STROBE) for observational studies [24, 25] and the Transparent Reporting of a Multivariable Prediction Model for Individual Prognosis or Diagnosis (TRIPOD) for prediction model development [26].

### Study setting

Data were extracted from the Malaysian National Stroke Registry (NSR), a nationwide, prospective hospital-based stroke registry initiated in 2009 under the National Neurology Registry (NNeuR) of the Ministry of Health Malaysia (http://app.acrm.org.my/nneur) [11, 22, 27]. Malaysia, located in Southeast Asia, comprises Peninsular Malaysia and East Malaysia, with a multi-ethnic population of approximately 32.78 million (2021 estimate) [28], and reflects a rich cultural diversity, including Malays, Chinese, Indians, and other ethnic groups.

### Study population

The study included adult patients (aged ≥ 18 years) with a confirmed diagnosis of first-ever acute ischemic stroke, enrolled in the NSR within the study period, and who had functional outcome data at three months post-discharge. Stroke diagnoses were classified using the 11th Revision of the International Statistical Classification of Diseases and Related Health Problems (ICD-11) [29].

A first-ever acute ischemic stroke was defined as the first lifetime stroke event, associated with a measurable neurological deficit lasting more than 24 h, attributable to a presumed ischemic etiology [30, 31]. Patients were excluded if they: (i) had transient ischemic attacks (TIAs) or hemorrhagic strokes, (ii) experienced recurrent strokes, (iii) were critically or terminally ill (e.g., malignancy), (iv) self-discharged against medical advice, or (v) had incomplete records with > 50% missing data.

### Study outcomes

The primary outcome of interest in this study was functional status, assessed using the modified Rankin Scale (mRS) at three (3) months post-hospital discharge. The mRS is a validated scale ranging from 0 (no symptoms) to 6 (death) [32]. For this study, the functional status was categorized as good functional status [mRS scores 0–2] and poor functional status or functional disability [mRS scores 3–5] [32, 33], excluding the mRS score of 6 (death). The functional disability used in this study was defined by poor functional status. The three-month follow-up period was chosen based on evidence that significant stroke recovery outcomes typically occur within this timeframe [34]. The mRS is widely used in stroke research and clinical practice due to its simplicity, reproducibility, and ability to assess functional outcomes across varying levels of stroke severity, including mild cases [35].

### Data collection

Data were collected by the researcher from the Malaysian stroke registry, including sociodemographic data (age, gender, and ethnicity), clinical characteristics such as Glasgow Coma Scale (GCS) (level of consciousness) [36], Oxfordshire Community Stroke Project (OCSP) classification of stroke subtypes (TACI: total anterior circulation infarct; LACI: lacunar infarct; PACI: partial anterior circulation infarct; POCI: posterior circulation infarct) [37], and National Institutes of Health Stroke Scale (NIHSS) (stroke severity) [38]. The GCS, as a complementary measure of consciousness, may provide additional prognostic value in conjunction with the NIHSS. The GSC is categorized into mild (13–15 points), moderate (9–12 points) and severe (1–8 points), while NIHSS is categorized into minor (1–4 points), moderate (5–15 points), moderate to severe (16–20 points), and severe stroke (21–42 points).

Other clinical variables collected include comorbidities (such as diabetes, hypertension, hyperlipidemia). Hypertension was defined as a systolic blood pressure ≥ 140 mmHg or a diastolic blood pressure ≥ 90 mmHg on repeated measurements [39], a self-reported history of hypertension, or the use of antihypertensive medications. Diabetes was defined as a fasting plasma glucose level ≥ 126 mg/dL (7.0 mmol/L), random plasma glucose ≥ 200 mg/dL (11.1 mmol/L) with classic hyperglycemia symptoms, HbA1c ≥ 6.5% [40], a self-reported history of diabetes, or the use of antidiabetic medications. Hyperlipidemia was defined as a total cholesterol level ≥ 240 mg/dL (6.2 mmol/L), low-density lipoprotein cholesterol (LDL-C) ≥ 160 mg/dL (4.1 mmol/L), high-density lipoprotein cholesterol (HDL-C) < 40 mg/dL (1.0 mmol/L) in men and < 50 mg/dL (1.3 mmol/L) in women, or triglycerides ≥ 150 mg/dL (1.7 mmol/L) based on NCEP ATP III guidelines [41], a self-reported history or use of lipid-lowering therapy.

In addition, to adjust for acute stroke treatments that could influence prognosis, the prognostic model included adherence to the nine guidelines recommended key performance indicators (KPIs) for acute ischemic stroke management in Malaysia [11, 27]. The KPIs are thrombolytic therapy, antiplatelet therapy within 48 h of admission, dysphagia screening, deep vein thrombosis (DVT) prophylaxis, anticoagulation for patients with atrial fibrillation (AF), discharge on antiplatelet therapy, discharge on statin medication, stroke education, and rehabilitation referral.

### Sample size and sampling

All eligible patients in the NSR meeting the inclusion criteria were included. Sample size estimation was based on the events per predictor variable (EPV) rule, requiring a minimum of 10 outcome events per predictor [42]. For 10 predictors, at least 100 events were deemed sufficient to minimize overfitting.

### Statistical data analyses

Statistical analyses were performed in accordance with the Statistical Analyses and Methods in the Published Literature (SAMPL) guidelines [43]. The analyses were conducted using IBM SPSS Statistics version 26.0 (IBM Corp., Armonk, NY, USA). Missing data were handled using multiple imputation methods. Descriptive statistics were presented as frequencies (percentages) for categorical variables and means (standard deviations) or medians (interquartile ranges, IQR) for continuous variables, depending on the data distribution. Normality was assessed using the Kolmogorov-Smirnov test and visual inspection of histograms. The models were built using binary logistic regression to analyze the primary outcomes (presence or absence of functional disability). Overall, a *p* ≤ 0.05 was considered statistically significant.

### Model development

Our prognostic models were developed using logistic regression due to its suitability for binary outcomes, interpretability in clinical settings, and ease of visualization [44, 45]. Although more complex machine learning algorithms have been applied in clinical prediction, evidence suggests that logistic regression often achieves comparable predictive accuracy, particularly with moderate-sized datasets and well-defined clinical variables [46, 47]. The models were developed using random sampling, and the data were split into a development cohort (*n* = 674; 75%) and an internal validation cohort (*n* = 225; 25%).

#### Univariable analysis

Simple logistic regression (SLR) was used to screen associations of functional disability with potential predictor variables, including sociodemographic data (age, gender, and ethnicity), clinical characteristics such as the GCS scores, NIHSS scores, OCSP classification stroke subtypes (TACI, POCI, OACI, and LACI), comorbidities (diabetes, hypertension, hyperlipidemia), and adherence to KPIs for acute ischemic stroke management [11, 27]. The KPIs include thrombolysis, antiplatelet therapy within 48 h., dysphagia screening, lipid-lowering therapy, antiplatelet therapy upon discharge, DVT prophylaxis, anticoagulation for AF, stroke education (self-care education, medication counseling), and rehabilitation (physical therapy care plan) and outcomes (presence or absence of functional disability). Variables with *p ≤* 0.25 or with established predictive value were included in the multivariable analysis [48].

#### Multivariable analysis

Backward stepwise multiple logistic regression (MLR) was employed to construct the final model. Model assumptions were assessed using the Hosmer–Lemeshow test, and Omnibus tests were used to evaluate overall model fit. Cox & Snell R² and Nagelkerke R² were used to estimate model variance [49]. Results were reported as adjusted odds ratios (aOR) with 95% confidence intervals (CI). A *p* ≤ 0.05 was considered statistically significant.

### Model validation

Internal validation was conducted using bootstrap resampling (*n* = 100 replications) to assess model performance and reduce overfitting [50–52]. Model calibration, as measured by the agreement between predicted and observed outcomes, was evaluated using the Hosmer–Lemeshow goodness-of-fit test. Patients were stratified into quintiles of predicted risk, and the calibration model’s intercept (ideal = 0) and slope (ideal = 1) were assessed [51]. Model discrimination, the ability to differentiate between those with and without functional disability, was assessed using the Receiver Operating Characteristic (ROC) curve. The Area Under the Curve (AUC) was reported, with values ranging from 0.5 (no discrimination) to 1.0 (perfect discrimination) [51, 53].

## Results

### Description of the study population

A total of 899 eligible patients with first-ever acute ischemic stroke were included in the study. The mean (± SD) age was 60.1 ± 10.8 years, with the majority aged 60 years and above. Most patients were males, 547 (60.8%), and predominantly of Malay ethnicity, 657 (73.1%). The OCSP classification revealed that most patients were diagnosed with LACI (351, 39.0%), followed by PACI (261, 29.0%), TACI (136, 15.1%), and POCI (104, 11.6%). The average GCS score was 14.1 ± 2.1, with most patients presenting with mild severity, 748 (83.2%), followed by moderate, 127 (14.1%). The stroke severity assessed using the NIHSS had an average score of 7.9 ± 7.3. The majority were classified as having moderate, 383 (42.6%), and mild, 309 (34.4%) strokes. Common comorbidities were found to be hypertension, 618 (68.7%), diabetes mellitus, 421 (46.8%), and hyperlipidemia, 192 (21.4%). Functional disability was observed in 484 patients (53.8%) at 3 months follow-up (Table 1).

**Table 1:** Characteristics of patients with first-ever acute ischemic stroke

Variable	Total, *n* (%) (*N* = 899)	Functional Disability, *n* (%) (*N* = 484; 53.8%)
Age (years)	61.1 (10.8)^**a**^	
Gender		
Female	352 (39.2)	206 (42.6)
Male	547 (60.8)	278 (57.4)
Ethnicity		
Malay	657 (73.1)	377 (77.9)
Chinese	128 (14.2)	60 (12.4)
India	22 (2.4)	16 (3.3)
Others	92 (10.2)	31 (6.4)
OCSP classification		
TACI	136 (15.1)	76 (15.7)
PACI	261 (29.0)	155 (32.0)
POCI	104 (11.6)	45 (9.3)
LACI	351 (39.0)	181 (37.4)
Unclassified	47 (5.2)	27 (5.6)
GCS score (1–15)	14.1 (2.1) ^**a**^	
Mild	748 (83.2)	14 (2.9)
Moderate	127 (14.1)	77 (15.9)
Severe	24 (2.7)	393 (81.2)
NIHSS score (0–42)	7.9 (7.3) ^**a**^	
None	71 (7.9)	37 (7.6)
Mild	309 (34.4)	149 (30.8)
Moderate	383 (42.6)	222 (45.9)
Moderate-Severe	69 (7.7)	32 (6.6)
Severe	67 (7.5)	44 (9.1)
Risk factors		
Hypertension	618 (68.7)	350 (72.3)
Diabetes	421 (46.8)	258 (53.3)
Hyperlipidemia	192 (21.4)	115 (23.8)
Ischemic heart disease	87 (9.7)	51 (10.5)
Atrial fibrillation	28 (3.1)	12 (2.5)
Hospital arrival (≤ 3 hours)	235 (26.1)	117 (24.2)

*SD* Standard deviation, *n* frequency, *N* total, *%* percentage, functional disability (modified Rankin Scale, mRS score 3–5), *GSC* Glasgow Coma Scale [mild (13–15 points), moderate (9–12 points) and severe (1–8 points)], *NIHSS* National Institute of Health Stroke Scale [minor (1–4 points), moderate (5–15 points), moderate to severe (16–20 points), and severe stroke (21–42 points)], *OCSP* Oxfordshire Community Stroke Project [*TACI* total anterior circulation infarct, *LACI* Lacunar infarct, *PACI* Partial anterior circulation infarct, *POCI* Posterior circulation infarct]

^a^: Mean (SD)

### Unadjusted predictors functional disability (univariable model)

Univariable analysis showed that increasing age (OR 1.02; 95% CI 1.01–1.03; *p* = 0.003) and female gender (OR 1.37; 95% CI 1.04–1.79; *p* = 0.024) were significantly associated with a higher risk of functional disability. Compared to the Malay ethnicity, Chinese (OR 2.65; 95% CI 1.67–4.19; *p* < 0.001) and other ethnic groups (OR 5.25; 95% CI 1.87–14.74; *p* = 0.002) had significantly higher risks of disability. Higher GCS scores were associated with lower risk of disability (OR 0.93; 95% CI 0.87–0.99; *p* = 0.027), while higher NIHSS scores were associated with increased risk of disability (OR 1.02; 95% CI 1.00–1.04; *p* = 0.017). Hypertension (OR 1.43; 95% CI 1.08–1.90; *p* = 0.013) and diabetes (OR 1.77; 95% CI 1.35–2.30; *p* < 0.001) were significantly associated with increased risk of functional disability.

Adherence to guideline-KPIs were found to significantly reduced the risk of functional disability, including: antiplatelet therapy within 48 h (OR 0.31; 95% CI 0.22–0.43; *p* < 0.001), dysphagia screening (OR 0.25; 95% CI 0.17–0.35; *p* < 0.001), antiplatelet therapy at discharge (OR 0.22; 95% CI 0.16–0.32; *p* < 0.001), lipid-lowering therapy (OR 0.57; 95% CI 0.43–0.75; *p* < 0.001), stroke education (OR 0.06; 95% CI 0.04–0.09; *p* < 0.001), and rehabilitation (OR 0.25; 95% CI 0.19–0.33; *p* < 0.001). (Table 2)

**Table 2:** Factors associated with functional disability [univariable model]

Variables	OR (95% CI)	*p*-value (*)
Age (years)	1.02 (1.01–1.03)	0.003
Gender (female)	1.37 (1.04–1.79)	0.024
Ethnicity		
Malay	1	
Chinese	2.65 (1.67–4.19)	< 0.001
India	1.74 (0.99–3.02)	0.051
Others	5.25 (1.87–14.74)	0.002
Stroke subtype		
LACI	1	
TACI	0.84 (0.57–1.25)	0.392
PACI	1.15 (0.76–1.76)	0.502
POCI	0.60 (0.36–1.01)	0.053
Unclassified	1.07 (0.55–2.08)	0.852
Hospital arrival (≤ 3 h)	0.82 (0.61–1.11)	0.198
GCS score (1–15)	0.93 (0.87–0.99)	0.027
NIHSS score (0–42)	1.02 (1.00-1.04)	0.017
Risk factors		
Hypertension	1.43 (1.08–1.90)	0.013
Diabetes	1.77 (1.35–2.30)	< 0.001
Hyperlipidemia	1.37 (0.99–1.89)	0.058
Ischemic heart disease	1.24 (0.79–1.94)	0.347
Atrial fibrillation	0.63 (0.30–1.36)	0.240
KPIs adherence		
Thrombolysis	0.73 (0.24–2.19)	0.577
Antiplatelet within 48 h.	0.31 (0.22–0.43)	< 0.001
Dysphagia screening	0.25 (0.17–0.35)	< 0.001
DVT prophylaxis	1.04 (0.77–1.40)	0.793
Antiplatelet upon discharge	0.22 (0.16–0.32)	< 0.001
Anticoagulant for AF	0.78 (0.51–1.21)	0.272
Lipid-lowering agent	0.57 (0.43–0.75)	< 0.001
Stroke education	0.06 (0.04–0.09)	< 0.001
Rehabilitation	0.25 (0.19–0.33)	< 0.001

*AF* Atrial fibrillation, *DVT* Deep vein thrombosis, *KPIs* Key performance indicators, functional disability (modified Rankin Scale, mRS score 3–5), *GSC* Glasgow Coma Scale [mild (13–15 points), moderate (9–12 points) and severe (1–8 points)], *NIHSS* National Institute of Health Stroke Scale [minor (1–4 points), moderate (5–15 points), moderate to severe (16–20 points), and severe stroke (21–42 points)], *OCSP* Oxfordshire Community Stroke Project [*TACI* Total anterior circulation infarct, *LACI* Lacunar infarct, *PACI* Partial anterior circulation infarct, *POCI* Posterior circulation infarct], *OR* Odds ratio, *CI* Confidence interval

*: Statistical significance at *p ≤* 0.05 using univariable logistic regression

### Adjusted predictors of functional disability (multivariable model)

Multivariable analysis revealed that increasing age (aOR 1.02; 95% CI 1.00–1.04; *p* = 0.024) and female gender (aOR 1.54; 95% CI 1.06–2.23; *p* = 0.024) were significant predictors of functional disability. Compared to patients with LACI, those with POCI had a significantly lower risk of disability (aOR 0.38; 95% CI 0.18–0.78; *p* = 0.009). Diabetes mellitus also remained a significant risk factor increasing the risk of disability (aOR 1.66; 95% CI 1.15–2.40; *p* = 0.007). However, adherence to KPIs were found to decrease the risk of disability, including: antiplatelet therapy within 48 h (aOR 0.48; 95% CI 0.28–0.80; *p* = 0.005), lipid-lowering therapy (aOR 0.56; 95% CI 0.37–0.83; *p* = 0.004), stroke education (aOR 0.09; 95% CI 0.05–0.16; *p* < 0.001), and rehabilitation (aOR 0.43; 95% CI 0.29–0.64; *p* < 0.001). (Table 3)

**Table 3:** Factors associated with functional disability [multivariable model]

Variables	β (SE)	aOR (95% CI)	*p*-values
Age (years)	0.020 (0.009)	1.02 (1.00-1.04)	0.024
Gender (female)	0.428 (0.190)	1.54 (1.06–2.23)	0.024
Stroke subtype			
LACI	1		
TACI	−0.139 (0.297)	0.87 (0.49–1.56)	0.641
PACI	−0.259 (0.307)	0.77 (0.42–1.41)	0.399
POCI	−0.973 (0.372)	0.38 (0.18–0.78)	0.009
Unclassified	−0.944 (0.554)	0.39 (0.13–1.15)	0.088
Risk factors			
Diabetes	0.507 (0.189)	1.66 (1.15–2.40)	0.007
KPIs adherence			
Antiplatelet in 48 hrs.	−0.739 (0.264)	0.48 (0.29–0.80)	0.005
Lipid-lowering therapy	−0.589 (0.202)	0.56 (0.37–0.83)	0.004
Stroke education	−2.428 (0.307)	0.09 (0.05–0.16)	< 0.001
Rehabilitation	−0.847 (0.204)	0.43 (0.29–0.64)	< 0.001
Intercept	3.318 (0.939)		

*KPIs* Key performance indicators, functional disability (modified Rankin Scale, mRS score 3–5), *OCSP* Oxfordshire Community Stroke Project [*TACI* Total anterior circulation infarct, *LACI* Lacunar infarct, *PACI* Partial anterior circulation infarct, *POCI* Posterior circulation infarct], *β* regression coefficient, *SE* Standard error, *aOR* adjusted odds ratio, *CI* Confidence interval

*: Statistical significance at *p* ≤ 0.05 using multivariable logistic regression

### Model validation for predicting functional disability

**Fig. 1:**
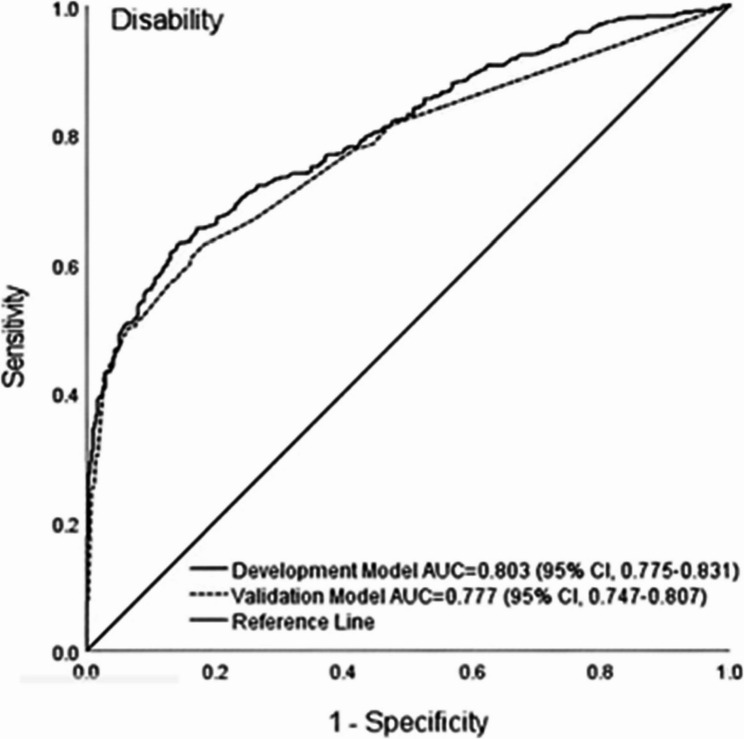
ROC curve validating the final model predicting functional disability. AUROC: Area under the receiver operating characteristics; AUC: Area under the curve; CI: Confidence interval

The prognostic models for functional disability demonstrated good performance in both the development and validation cohorts. The AUC was 0.803 (95% CI 0.78–0.83) for the development model and 0.777 (95% CI 0.75–0.81) for the validation model. Model calibration was satisfactory, as indicated by non-significant Hosmer-Lemeshow test results (development model: χ² = 7.61, *p* = 0.473; validation model: χ² = 0.94, *p* = 0.967). The models demonstrated good overall fit (Omnibus test: *p* < 0.001), with Nagelkerke R² values of 0.402 (development) and 0.363 (validation). (Table 4; Fig. 1)

**Table 4:** Model validation for predicting functional disability

Performance measures	Models	
	Development	Validation
Discrimination (*AUC*)^a^	0.803 (0.78–0.83)	0.777 (0.75–0.81)
Calibration (*H-L*)	*X*^2^ = 7.61 (*p* = 0.473)	*X*^2^ = 0.94 (*p* = 0.967)
Omnibus test (*Goodness-of-fit*)	*X*^2^ = 247.18 (*p <* 0.001)	*X*^2^ = 65.37 (*p <* 0.001)
Nagelkerke R square (*R*^*2*^)	0.402	0.363

^a^: *CI* 95% confidence interval, *AUC* Area under the curve, *H-L* Hosmer-Lemeshow test, X^2^ Chi-square test

## Discussion

This study developed and validated a prognostic model of functional disability following a first-ever acute ischemic stroke using data from a Malaysian stroke cohort. The models demonstrated robust predictive performance, with high discriminative ability, good calibration, and consistent reliability. These results mark a substantial contribution to stroke prognostication literature, particularly in settings where predictive models that incorporate adherence to clinical guidelines are scarce. Our findings demonstrate the potential clinical utility of integrating patient characteristics and quality-of-care indicators in predicting post-stroke outcomes, supporting more individualized and timely interventions.

The final multivariable model identified several independent predictors of functional disability: age, female gender, OCSP stroke subtype, diabetes, and adherence to guideline-based KPIs. Age remains a well-documented determinant of stroke outcomes. As physiological reserve declines with aging, recovery potential diminishes, and comorbidities often complicate rehabilitation. This aligns with global evidence highlighting older age as a predictor of poorer post-stroke function [54]. Gender disparities were also evident, with female patients experiencing worse outcomes. This may be attributed to several biological and social factors, including postmenopausal hormonal changes, longer life expectancy, and pre-stroke dependency [55]. These findings emphasize the importance of sex-specific interventions and recovery pathways.

Stroke subtype emerged as a strong predictor, with posterior circulation infarcts (POCI) associated with better functional outcomes compared to total anterior circulation infarcts (TACI), which often result in extensive cortical damage and more severe neurological deficits [56, 57]. The inclusion of diabetes further reinforces the impact of vascular risk burden on stroke prognosis. Hyperglycemia is known to exacerbate infarct size and impair neuronal recovery, resulting in poorer functional outcomes [58–60]. This finding highlights the urgent need for integrated post-stroke management strategies addressing glycemic control and vascular risk reduction. Effective management strategies, including timely interventions, rehabilitation programs, and risk factor modification, are crucial in reducing the incidence and severity of functional impairments.

A unique and pivotal feature of this study was the inclusion of adherence to guideline-recommended KPIs as predictive variables. These included early administration of antiplatelet therapy, lipid-lowering therapy, stroke education, and rehabilitation services. Each intervention independently reduced the likelihood of functional disability, underscoring the importance of guideline-concordant care. These results are consistent with prior studies demonstrating improved functional outcomes and survival among patients receiving evidence-based stroke interventions [61]. Our findings support the implementation of integrated stroke care pathways that ensure adherence to established clinical guidelines. Strengthening adherence through audit-feedback systems, decision support tools, and workforce training could yield substantial gains in functional recovery and overall care quality.

A study in acute ischemic stroke patients from controlled trials found that a simple model using age and NIH Stroke Scale score accurately predicted 3-month survival and recovery, but its generalizability to the uncontrolled broader stroke population is limited [62]. The predictive accuracy of our models compares favorably with other established prognostic tools, such as the iScore [63], ASTRAL score [64], and THRIVE score [65]. However, unlike these tools, our models uniquely integrate both clinical and process-of-care factors, enhancing their relevance to real-world acute stroke management. The inclusion of KPI adherence reflects not just patient-level risks but also system-level performance, making the model more adaptable for use in quality improvement.

The findings of this study have significant implications for clinical practice, public health, and healthcare policy. From a clinical standpoint, the validated model can facilitate early risk stratification and guide the treatment process related to KPIs. For instance, clinicians could use the model to identify high-risk patients upon admission and tailor intensive treatment and rehabilitation referrals. Web-based or EMR-integrated tools derived from this model could streamline decision-making in stroke units. At the public health level, the model reinforces the importance of population-level adherence to evidence-based care practices. It also highlights the value of national stroke registries in generating actionable insights to optimize care delivery and monitor healthcare system performance. This study benefits from a large, homogeneous sample of patients with first-ever acute ischemic stroke, enabling precise estimation of outcomes while minimizing confounding due to prior stroke history. The use of a national stroke registry enhances generalizability within Malaysia and reflects routine clinical practice.

However, limitations must be acknowledged. First, the reliance on administrative data may affect the completeness and granularity of some variables. Second, the study was restricted to a single country (Malaysia), and findings may not be directly generalizable to other populations, such as those in Western or Middle Eastern settings, without external validation. Finally, functional disability was assessed at a three-month follow-up post-discharge; longer-term functional outcomes (e.g., at 12 months to several years) were not evaluated, which could provide a more comprehensive picture of recovery trajectories. In addition, the study’s predictions are based on treatment information at baseline, raising concerns about their temporal relevance and representativeness. The study employed the OCSP classification rather than the more widely used TOAST classification due to its clinical significance and alignment with the records available in the Malaysian stroke registry. Additionally, other clinically relevant variables, such as BMI and smoking status, could not be included. The very small sample that received thrombolysis may have resulted in selection bias and inadequate control, leading to exclusion from the final model.

Future studies should aim to externally validate these models in diverse geographic and healthcare settings, particularly in low- and middle-income countries where stroke burden is high, but resources are limited. Additionally, research should explore the real-time integration of such models into clinical workflows and assess their impact on decision-making, resource use, and patient outcomes. Evaluating long-term predictive validity and incorporating patient-reported outcome measures could further enhance the utility and comprehensiveness of future models.

## Conclusion

This study developed and validated a prognostic model of functional disability following a first-ever acute ischemic stroke using the Malaysian stroke cohort. The model demonstrated good predictive performance, with excellent calibration. By integrating both patient-level risk factors and adherence to evidence-based care practices, the model offers a practical tool for early risk stratification, personalized care planning, and improved decision-making in acute stroke management. Furthermore, the findings highlight the vital role of national stroke registries in advancing stroke research and care, reinforcing the need to sustain and strengthen such data-driven initiatives to support high-quality, guideline-informed stroke services.

## Data Availability

The datasets supporting the conclusions of this article are included within the article. The datasets used and/or analyzed during the current study are available from the corresponding author on reasonable request.
